# Hepatitis B virus (HBV) genotypes in Egyptian pediatric cancer patients with acute and chronic active HBV infection

**DOI:** 10.1186/1743-422X-4-74

**Published:** 2007-07-15

**Authors:** Abdel-Rahman N Zekri, Mohamed M Hafez, Nahed I Mohamed, Zeinab K Hassan, Manal H El-Sayed, Mohsen M Khaled, Tarek Mansour

**Affiliations:** 1 Virology and Immunology Unit, Cancer Biology Department, National Cancer Institute, Cairo University, 1st Kasr El-Aini st, Cairo, Egypt; 2 Microbiology Department, Faculty of Medicine, Suez Canal University, Ismailia, Egypt; 3 Pediatric Department, Faculty of Medicine, Ain Shams university, Abbasya, Cairo, Egypt; 4 National Diabetes Institute, Ministry of Health, Egypt, 1st Kasr El-Aini st., Cairo, Egypt

## Abstract

**Background:**

There are eight genotypes of hepatitis B virus (A-H) and subgenotypes are recognized. Genotyping can be accomplished based on a partial sequence of HBV genome such as the pre-S or S gene. Several methods have been developed and used for HBV genotyping. This study was undertaken to determine the HBV genotypes in Egyptian pediatric cancer patients with acute and chronic liver disease.

**Methods:**

HBV genotypes were determined in 22 patients who had acute forms of liver disease (AH) and in 48 patients with chronic active hepatitis (CAH). A type-specific primer based the nested-PCR method was employed in the HBV genotyping.

**Results:**

This study showed that HBV infections in pediatric cancer patients are attributed predominantly to viral genotypes D and B that constituted 37.1% and 25.7%, respectively of the total infections. In addition, there was a relatively high prevalence of mixed infections of 15.7% among the studied group especially mixed A/D genotype infections. Genotype D was found significantly more often in patients with CAH than in patients with AH [23/48(47.9%) *v *3/22 (13.6%)].

**Conclusion:**

These findings show the distribution of HBV A-D genotypes in pediatric cancer Egyptian patients. Furthermore, our results indicate a markedly high prevalence of mixed A/D genotype infections in subjects with CAH and a possible association of mixed infections with the severity of liver diseases.

## Background

HBV infection is very common worldwide, and more than 350 million people are chronic carriers [1]. HBV infection is associated with different clinical pictures and leads to chronic carrier state in 5 to 10% patients infected in adult life and 85 to 90% of those infected in infancy [2]. Infection with HBV can also lead to progressive liver disease, including liver cirrhosis and hepatocellular carcinoma (HCC) with approximately 1 million HBV-associated deaths from HCC every year [3].

HBV was formerly classified into four different subtypes that were afterward subdivided according to the antigenic determinants of HBsAg in *adw *(*adw2 *and *adw4*), *ayw *(*ayw1*, *ayw2*, *ayw3*, and *ayw4*), *adr *(*adrq+ adrq-*), and *ayr *[4].

Another classification reflecting the phylogenetic origin of the virus isolates was later proposed dividing HBV into six genotypes designated A to F. These genotypes were differentiated by a sequence divergence in the entire genome exceeding 8% [5]. Seventh and eighth genotypes were reported: genotype G, which has an insertion of 36 nucleotides (nt) in the core gene and was discovered in France and United States [6], and genotype H, which was found in Nicaragua, Mexico, and California and has probably split off from genotype F within the New World [7].

Therefore, eight genotypes of HBV (A-H) are currently recognized, and subgenotypes have recently been described which differ by at least 4% [8]. The genotypes show a distinct geographical distribution between and even within regions, and are proving to be an invaluable tool in tracing the molecular evolution and patterns and modes of spread of HBV. Structural and functional differences between genotypes can influence the severity, course and likelihood of complications, hepatitis B e antigen (HBeAg) seroconversion and response to treatment of HBV infection and possibly vaccination against the virus [9]. Although the number of studies on these genotypes has increased dramatically during recent years, much remains to be learnt about their full implications.

Africa is one of the highly endemic regions of HBV, with 5 genotypes A-E identified. Genotype A in Kenya [10], genotype D in Tunisia [11], genotype A-D in South Africa [12] and genotype E in Nigeria [13] were reported as predominant genotypes in these countries. Apart from these reports, however, there is little information of genotype distribution in Africa despite the importance of this infection in this region.

Few reports described the frequency of HBV genotypes in Egypt. In one study, the genotypes of HBV isolated from 105 serum samples from Egyptian carriers were determined by sequencing and found that HBV genotype D are most prevalent in Egypt [14]. Naito *et al. *(2001) examined 2 serum samples positive for HBV DNA by primer specific PCR and these turned to be of genotype D [15].

HBV genotyping may evolve from a research tool into being an essential clinical diagnostic test, very much as HCV genotyping did. One hurdle in the introduction of HBV genotyping to clinical practice is the lack of a simple, rapid, and accurate test [16]. Currently, HBV genotypes can be determined by several methods, including direct sequencing [6], restriction fragment length polymorphism analysis [17], line-probe assay [18], PCR using type-specific primers [15], colorimetric point mutation assay [19], ligase chain reaction assay [20], and enzyme-linked immunosorbent assay for genotype-specific epitopes [10]. Direct sequencing is the most accurate and permits detection of the common as well as uncommon mutations but is also the most expensive and tedious [16]. Development of rapid, simple, and standardized assays that can detect all known genotypes can accelerate progress in research on the clinical significance of HBV genotypes.

The aim of this study was to investigate the frequency of HBV genotypes in Egyptian patients by PCR using type-specific primers.

## Patients and methods

### Patients

The study was approved by ethical committee and informed consents were obtained from all parents of each patients participating in the study. This study included 70 pediatric cancer patients (38 males and 32 females) attending the National Cancer Institute (NCI), Cairo University suffering from hepatitis and were diagnosed as HBV infection. HBV was diagnosed based on clinical data, liver function tests, HBV serum markers and HBV DNA by PCR. Serum samples were collected from patients in a period between December 2005 to January 2007 and stored at -20°C until used.

The age of the patients ranged from 3 years to 18 years (mean, 10.5). The study group comprised 22 patients with acute hepatitis B infection (AH) and 48 with chronic active hepatitis (CAH). HBV related acute forms of liver disease were diagnosed based on the appearance of hepatitis B surface antigen (HBsAg) and the presence of anti-HBc-IgM. Patients who had HBsAg for more than 6 months with an abnormal alanine aminotransferase (ALT) level and the presence of anti-HBc-IgG were diagnosed as CAH. All patients were HBeAg positive.

### Serological markers

Serologic markers for HBV (hepatitis B surface antigen [HBsAg], hepatitis Be antigen [HBeAg] and antibodies to hepatitis B core antigen [anti-HBc]) (enzyme immunoassay [EIA]; Adaltis, Italy) infection were detected with current standard assay. All serologic assays were carried out according to the manufacturer's instructions.

### DNA extraction

The QIAamp DNA extraction kit (QIAGEN GmbH, Hilden Germany) was employed for DNA extraction from serum samples according to the manufacturer's instructions.

### Serum HBV DNA detection

**Figure 1:**
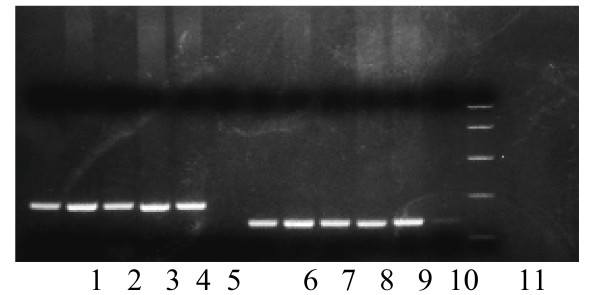
DNA amplification by PCR using conserved nature of nucleotide sequences in regions of the pre-S1 through S genes followed by gel electrophoresis, ethidium bromide staining lanes 1–5 represent positive cases for genotype C, lanes 6–10 represent positive cases for genotype D and lane 11 is PCR marker (Promega, Madison, Wis. USA).

All DNA extracts were analyzed for HBV genomes with polymerase chain reaction (PCR) assays to detect the core genes, according to previously described methods [21]. Briefly, 100 μl of reaction mixture containing 10 μl of extracted DNA, 50 mM potassium chloride, 10 mM TRIS-hydrochloric acid (pH 8.3), 2 mM magnesium chloride, 200 μM deoxyribonucleosides, 2.5 U of *Taq *polymerase (Perkin-Elmer Cetus, Norwalk, Conn.), and 20 pmol each of the oligonucleotide primers C1 sense CTGGGAGGAGTTGGGGGA (1730–1747) and C2 antisense GTAGAAGAATAAAGCCC (2503–2487) for the core gene. Amplification was performed for one cycle at 95°C for 5 min followed by 35 cycles, each consisting of denaturing for 1 min. at 94°C, annealing for 1 min at 55°C, and extension for 1.5 minutes at 72°C. The amplification products were visualized on an ethidium bromide-stained 2% agarose gel (fig 1).

### Genotype analysis

A genotyping system based on PCR using type-specific primers was used in this study for the determination of genotypes A through F of hepatitis B virus according to previously described methods by Naito *et al*. 2001[15]. The sequences of PCR primers used in this study are shown in Table 1. The first round PCR primers and second-round PCR primers were designed on the basis of the conserved nature of nucleotide sequences in regions of the pre-S1 through S genes, irrespective of the six HBV genotypes [15]. P1 (sense) and S1-2 (antisense) were universal outer primers (1,063 bases). B2 was used as the inner primer (sense) with a combination called mix A for genotypes A, B, and C. Mix A consisted of antisense primers BA1R (type A specific), BB1R (type B specific), and BC1R (type C specific). B2R was used as the inner primer (antisense) with a combination called mix B for genotypes D, E, and F. Mix B consisted of sense primers BD1 (type D specific), BE1 (type E specific), and BF1 (type F specific). These primer combinations for second-round PCR were designed on the basis of the differences in the sizes of the genotype-specific bands. The type-specific primers were designed on the basis of the conserved nature of those sequences within a genotype and on the basis of their poor homology with the sequences derived from other HBV genotypes [15]. The first PCR was carried out in 40 ul of a reaction mixture containing 100 ng of each outer primer, a 200 mM concentration of each of the four deoxynucleotides, 2.5 U of Taq DNA polymerase (Promega, France) 1× PCR buffer containing containing (50 mM KCl, 10 mM Tris pH 8.3) and 1.5 mM MgCl2. The thermocycler (Eppendorf, Germany) was programmed to first incubate the samples for 5 min at 95°C, followed by 40 cycles consisting of 94°C for 1 min, 55°C for 1 min and 72°C for 2 min. Two second-round PCRs were performed for each sample, with the common universal sense primer (B2) and mix A for types A through C and the common universal antisense primer (B2R) and mix B for types D through F. A 1 ml aliquot of the first PCR product was added to two tubes containing the second sets of each of the inner primer pairs, each of the deoxynucleotides, Taq DNA polymerase, and PCR buffer, as in the first reaction. These were amplified for 40 cycles with the following parameters: preheating at 95°C for 5 min, 30 cycles of amplification at 94°C for 1 min, 58°C for 1 min, and 72°C for 1.30 min Genotypes of HBV for each sample were determined by identifying the genotype-specific DNA bands. The two different second-round PCR products from one sample were visualized on an ethidium bromide-stained 3% agarose gel. The sizes of PCR products were estimated according to the migration pattern of a 50 lane PCR marker (Promega, Madison, WIs.).

**Table 1:** 

**Primer**	**Sequence ^*a*^(position, specificity, and polarity)**
**First PCR**	
P1	5'-TCA CCA TAT TCT TGG GAA CAA GA-3' (nt 2823–2845, universal, sense)
S1-2	5'-CGA ACC ACT GAA CAA ATG GC-3' (nt 685–704, universal, antisense)
**Second PCR**	
**Mix A**	
B2	5'-GGC TCM AGT TCM GGA ACA GT-3' (nt 67–86, types A to E specific, sense)
BA1R	5'-CTC GCG GAG ATT GAC GAG ATG T-3' (nt 113–134, type A specific, antisense)
BB1R	5'-CAG GTT GGT GAG TGA CTG GAG A-3' (nt 324–345, type B specific, antisense)
BC1R	5'-GGT CCT AGG AAT CCT GAT GTT G-3' (nt 165–186, type C specific, antisense)
**Mix B**	
BD1	5'-GCC AAC AAG GTA GGA GCT-3' (nt 2979–2996, type D specific, sense)
BE1	5'-CAC CAG AAA TCC AGA TTG GGA CCA-3' (nt 2955–2978, type E specific, sense)
BF1	5'-GYT ACG GTC CAG GGT TAC CA-3' (nt 3032–3051, type F specific, sense)
B2R	5'-GGA GGC GGA TYT GCT GGC AA-3' (nt 3078–3097, types D to F specific, antisense)

^*a *^An "M" represents a nucleotide that could be either an A or a C; a "Y" represents a nucleotide that could be either a C or a T. nt: nucleotide.

### Statistical analysis

Analysis of data was carried out with the aid of SPSS package version 10.0. Parameters were compared using the Chi-square test. *P *values less than 0.05 were considered statistically significant.

## Results

### Distribution of HBV genotypes

**Figure 2:**
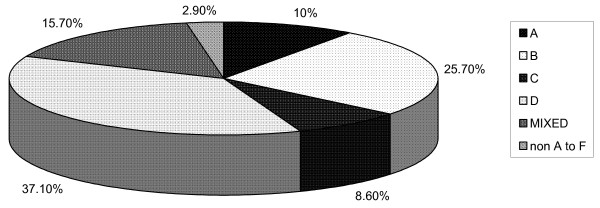
HBV genotype distribution in the studied group.

This study showed that HBV infections in pediatric cancer patients are attributed predominantly to viral genotypes D and B that constituted 37.1% and 25.7%, respectively of the total infections. In addition, there was a relatively high prevalence of mixed infections of 15.7% among the studied group. HBV genotypes A and C infections were the least observed and constituted 10% and 8.6% respectively of the total infections (fig 2). Two cases were not confined to any of the six genotypes studied (2.9%). No HBV genotype E or F was found in our study and furthermore, genotypes G and H were not determined.

### Association between liver disease and the prevalence of HBV genotypes

The distribution of HBV genotypes in the various forms of liver disease is shown in table 2. Among the 22 patients who had an acute form of liver disease, genotype B showed a predominance over the other genotypes with the following values respectively: 2 cases (9%) genotype A, 10 (45.5%) genotype B, 3 (13.6%) genotype C and 3 (13.6%) genotype D. In subjects with CAH (48 cases), the distribution of genotype A, B, C and D infections were as follows: 5 (10.4%), 8 (16.7%), 3 (6.3%), 23 (47.9%) respectively, with a predominance of genotype D. Genotype D was found significantly more often in patients with CAH than in patients with AH [23/48(47.9%) *v *3/22 (13.6%)]. Also, genotype A tended to be found more often in patients with CHD than in patients with AH [5/48 (10.4%) *v *2/22 (9%)] (*P *< 0.05).

**Table 2:** 

**Subjects N = 70**	**HBV genotypes**				
	
	**A**	**B**	**C**	**D**	**mixed**
**Acute hepatitis (22)**	2 (9%)	10 (45.5%)	3 (13.6%)	3 (13.6%)	4 (18.2%)
Chronic* active hepatitis (48)	5 (10.4%)	8 (16.7%)	3 (6.3%)	23 (47.9%)	7 (14.6%)

**Total**	7 (10%)	18(25.7%)	6 (8.6%)	26 (37.1%)	11 (15.7%)

*Two CAH cases were non A to F

### Distribution of mixed HBV genotypes

**Figure 3:**
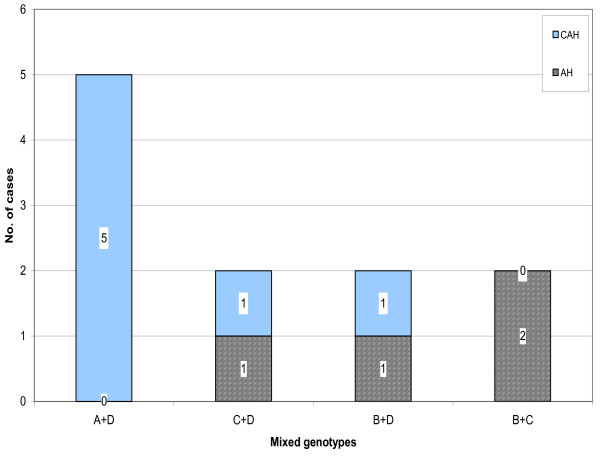
Distribution of mixed genotypes among the study groups.

Another finding in this study was the presence of 11 cases (15.7%) with mixed genotype infections. Five cases had both genotypes A and D, 2 cases had C and D, 2 other cases had B and D and 2 cases had genotypes B and C. The distribution of mixed genotypes among AH and CAH patients is shown in figure 3. CAH patients showed a higher prevalence of mixed genotypes than AH patients (7/11 (63.3%) *v *4/11 (36.3%) (Table 2). The comparative mixed infections between the two groups were statistically insignificant.

## Discussion

HBV is a typical example of a virus that attracts attention with its different genotypes, showing special geographic distribution around the world. A genetic classification based on the comparison of complete genomes has defined eight genotypes of HBV, which were designated from A through H [5-7]. Genotype D appears to predominate in the Mediterranean basin and the Middle East, and this is consistent with Egypt's geographical location in the world.

The genotyping of HBV is important to clarify the route and pathogenesis of the virus. In particular, the examination of sequence diversity among different isolates of the virus is important, because variants may differ in their patterns of serologic reactivity, pathogenicity, virulence, and response to therapy [4,22].

Several methods have been developed and used for HBV genotyping including direct sequencing [6], PCR based restriction fragment length polymorphism [17], line probe assay [18] and enzyme-linked immunoassay [10]. Recently a new genotyping method, based on PCR amplification assay using type-specific primers, which can identify all six major genotypes has been developed by Naito *et al. *[15]. We employed this handy, convenient and novel type-specific primer based PCR method in this study to investigate the prevalence of HBV genotypes in Egyptian pediatric cancer patients concomitantly infected with HBV.

Few reports described the frequency of HBV genotypes in Egypt. In this study, genotype D was reported as the predominant HBV genotype in the study subjects (37.1%) followed by genotype B that constituted 25.7%. These figures are in conformity with 2 other studies done in Egypt. In one study, the genotypes of HBV isolated from 105 serum samples from Egyptian carriers were determined by sequencing and found that HBV genotype D are most prevalent in Egypt [14]. Naito *et al. *(2001) examined 2 serum samples positive for HBV DNA by primer specific PCR and these turned to be of genotype D but they didn't find other genotypes as they only examined 2 serum samples [15].

Another similar study was done in Turkey, a country in the Middle East, to determine the hepatitis B virus genotypes in Turkish patients with chronic liver disease. Their study comprised 25 pediatric and 29 adult patients with chronic hepatitis B virus infection and genotype D was the dominant genotype in all of their cases [23].

Genotype D was found significantly more often in patients with CAH than in patients with AH (*P *< 0.05). Previous reports have indicated that genotype C was the predominant genotype in subjects with advanced chronic hepatitis (CH), liver cirrhosis (LC) and hepatocelluler carcinoma (HCC) [24,25] in contrast to our results. However, the number of patients for each pattern was insufficient to reach firm conclusions. Two reports from India have provided conflicting results in this context. In a study of chronic carriers in which, surprisingly, 50% of the patients were infected with genotype A strains and 50% were infected with genotype D strains, Thakur *et al. *[26] found that patients infected with genotype D strains had more severe chronic liver disease. In contrast, Gandhe *et al. *[27] could not find that genotype D influenced the outcome of chronic HBV infection in Indian patients. In another study, Ljunggren *et al. *[3] found that HBV genotype D may be associated with more active disease.

The distribution of HBV genotype B in acute forms of liver disease was higher than that seen in chronic forms of the disease suggesting an association of genotype B with more severe acute forms of liver disease. A similar result was observed in the study of Imamura *et al. *[28] that showed that genotype B was more prevalent in patients with FH and AH. They attributed this result to the possibility that Genotype B virus may have the motifs that strongly bind to HLA class I molecules, thereby resulting in activation of a stronger immune response and a more liver damage.

Double infections with two different HBV genotypes have been known since typing was done serologically [29]. Subsequently, evidence of super infection with HBV isolates of the same or different genotype was described in chronic HBV patients [30]. Super infection was accompanied by acute exacerbation of the chronic disease. Using different methods for genotyping, several reports described high rates of double infection with two different HBV genotypes in all parts of the world. Using these methods double infections have been found in a range from 4.4% [31] to 17.5% [32] of HBV infected patients. Even triple infections with HBV of genotype A, B and C have been described in 0.9% of HBV infected intravenous drug users [32].

In this study, we reported a prevalence of mixed genotype infections in study subjects of 15.7% especially those with CAH. HBV genotype A and D mixed infections accounted for 45.5% of the total mixed infections. We conclude that HBV mixed genotype infections could probably be of clinical significance in HBV-induced liver diseases. In light of the prevalence of mixed A/D genotype infections in our study, we propose that greater attention be paid to A/D mixed genotype screening in the management of HBV-induced chronic liver diseases and evaluation of therapy.

We therefore suggest that HBV genotyping become a routine exercise in clinical medicine and molecular epidemiology. As genotypes have different biological and epidemiological behavior, their detection and monitoring is more than just academic but also medically significant. Furthermore, efforts to prevent mixed infections (super-infection or co-infections) in patients with chronic hepatitis B should not be overlooked, especially in areas endemic for HBV infection. Since a small number of subjects were employed in our investigation, we propose that large scale studies be conducted to substantiate our findings. Such studies could also provide more insight into the association between co-infection and disease exacerbations as well as shed lighter on the molecular, virological and host mechanisms underlying the pathogenesis of HBV-related disease.

## Competing interests

The author(s) declare that they have no competing interests.

## Authors' contributions

A-R N Z: Conceived of the study, participated in its design and coordination, drafted the manuscript and coordinate the whole work team.

M M H: Sample collection, carried out the molecular genotyping studies, participated in the drafted the manuscript and performed the statistical analysis.

N I M: participated in the editing of the manuscript.

Z K H: sample collection and carried out the serological assays.

M H E: Responsible for the patient treatment and clinical data collection

M M K: participated in the editing the manuscript and clinical data.

T M: Coordinated the research effort.

All eight co-authors read and approved the final manuscript.
